# Keratinocyte-derived small extracellular vesicles supply antigens for CD1a-resticted T cells and promote their type 2 bias in the context of filaggrin insufficiency

**DOI:** 10.3389/fimmu.2024.1369238

**Published:** 2024-03-22

**Authors:** Adrian Kobiela, Weronika Hewelt-Belka, Joanna E. Frąckowiak, Natalia Kordulewska, Lilit Hovhannisyan, Aleksandra Bogucka, Rachel Etherington, Artur Piróg, Irena Dapic, Susanne Gabrielsson, Sara J. Brown, Graham S. Ogg, Danuta Gutowska-Owsiak

**Affiliations:** ^1^ Laboratory of Experimental and Translational Immunology, Intercollegiate Faculty of Biotechnology of the University of Gdańsk and the Medical University of Gdańsk, Gdańsk, Poland; ^2^ Department of Analytical Chemistry, Faculty of Chemistry, Gdańsk University of Technology, Gdańsk, Poland; ^3^ Department of Biochemistry, Faculty of Biology and Biotechnology, University of Warmia and Mazury, Olsztyn, Poland; ^4^ The Mass Spectrometry Laboratory, Intercollegiate Faculty of Biotechnology of University of Gdańsk and Medical University of Gdańsk, Gdańsk, Poland; ^5^ MRC Human Immunology Unit, NIHR Biomedical Research Centre, Radcliffe Department of Medicine, University of Oxford, Oxford, United Kingdom; ^6^ International Centre for Cancer Vaccine Science, University of Gdańsk, Gdańsk, Poland; ^7^ Division of Immunology and Allergy, Department of Medicine Solna, Karolinska Institutet, Stockholm, Sweden; ^8^ Department of Clinical Immunology and Transfusion Medicine, Karolinska University Hospital, Stockholm, Sweden; ^9^ Centre for Genomic and Experimental Medicine, Institute of Genetics and Cancer, University of Edinburgh, Edinburgh, United Kingdom

**Keywords:** CD1a, sEV, exosome, T cell, atopic dermatitis, filaggrin, allergic inflammation

## Abstract

**Introduction:**

Exosome-enriched small extracellular vesicles (sEVs) are nanosized organelles known to participate in long distance communication between cells, including in the skin. Atopic dermatitis (AD) is a chronic inflammatory skin disease for which filaggrin (*FLG*) gene mutations are the strongest genetic risk factor. Filaggrin insufficiency affects multiple cellular function, but it is unclear if sEV-mediated cellular communication originating from the affected keratinocytes is also altered, and if this influences peptide and lipid antigen presentation to T cells in the skin.

**Methods:**

Available mRNA and protein expression datasets from filaggrin-insufficient keratinocytes (shFLG), organotypic models and AD skin were used for gene ontology analysis with FunRich tool. sEVs secreted by shFLG and control shC cells were isolated from conditioned media by differential centrifugation. Mass spectrometry was carried out for lipidomic and proteomic profiling of the cells and sEVs. T cell responses to protein, peptide, CD1a lipid antigens, as well as phospholipase A2-digested or intact sEVs were measured by ELISpot and ELISA.

**Results:**

Data analysis revealed extensive remodeling of the sEV compartment in filaggrin insufficient keratinocytes, 3D models and the AD skin. Lipidomic profiles of shFLGsEV showed a reduction in the long chain (LCFAs) and polyunsaturated fatty acids (PUFAs; permissive CD1a ligands) and increased content of the bulky headgroup sphingolipids (non-permissive ligands). This resulted in a reduction of CD1a-mediated interferon-γ T cell responses to the lipids liberated from shFLG-generated sEVs in comparison to those induced by sEVs from control cells, and an increase in interleukin 13 secretion. The altered sEV lipidome reflected a generalized alteration in the cellular lipidome in filaggrin-insufficient cells and the skin of AD patients, resulting from a downregulation of key enzymes implicated in fatty acid elongation and desaturation, i.e., enzymes of the ACSL, ELOVL and FADS family.

**Discussion:**

We determined that sEVs constitute a source of antigens suitable for CD1a-mediated presentation to T cells. Lipids enclosed within the sEVs secreted on the background of filaggrin insufficiency contribute to allergic inflammation by reducing type 1 responses and inducing a type 2 bias from CD1a-restricted T cells, thus likely perpetuating allergic inflammation in the skin.

## Introduction

CD1a-mediated T-cell responses to lipid antigens have been identified as important players in skin inflammation in atopic dermatitis (AD), with both *ex vivo* and animal models indicating the importance of this antigen presentation pathway and its potential as a therapeutic target (1–6). The lipids contained within microbes have been shown to stimulate protective responses (7). Low levels of autoreactivity can also be observed in self-lipids, which are believed to be crucial for skin homeostasis and tissue integrity (8). The role of phospholipase A2 (PLA2) in the CD1a-T cell axis has also been recognized. By cleaving membrane phospholipids, this enzyme generates neoantigens that are suitable for CD1a binding. PLA2 may be expressed endogenously in the skin (9, 10), but PLA2 activity in exogenous sources, such as pathogens, insect venom, or house dust mites, also contributes to specific T-cell responses in allergy.

Small extracellular vesicles (sEVs), enriched in exosomes, are secreted organelles that fall within the 30 nm–150 nm size range and are released by all nucleated cells, including keratinocytes (11–16). Due to their unique biogenesis pathway, exosome-enriched sEVs acquire distinct characteristics that enable them to penetrate cells and enter the systemic circulation without damage. This, together with a set of cell type-dependent membrane receptors and specific molecular cargo, provides the basis for their involvement in long-distance communication, which includes messaging to the immune system. Previously, we identified a crucial role of keratinocyte-derived sEVs in a mechanism supporting epidermal homeostasis (16) and discovered how AD-relevant pathogens, *S. aureus* (16) and *C. albicans* (15), hijack sEV to safeguard their own growth on the skin. However, whether sEVs can contribute to T-cell responses in AD skin is not yet clear, and reports are scarce and inconclusive (11).

Importantly, to date, no data are available on the involvement of sEVs in lipid-specific CD1-specific T-cell immune responses, either in physiology or pathology, and we decided to investigate this in the context of the disease. Since loss-of-function mutations in the gene encoding the critical skin barrier protein, filaggrin (*FLG*), provides the strongest genetic predisposition for the disease and additional allergic manifestations in patients (17–20), we chose filaggrin insufficiency as a model of the disease. Filaggrin is an essential factor supporting skin barrier formation, from the structural function through to control of keratinocyte life cycle and differentiation (21–25) and this is reflected in abnormal functional properties of filaggrin-insufficient cells (23–25). The protein plays a critical role in skin immunity, supporting the mechanisms of innate defense (26–28), and has been previously shown to modulate dendritic cell capacity to present antigens (5, 29). We also showed that filaggrin insufficiency affects T-cell responses to peptides induced by activated keratinocytes (30); the protein itself also directly inhibits CD1a lipid neoantigen generation by PLA2 (5).

Here, we integrated data from filaggrin-insufficient cells cultured *in vitro*, and 3D organotypic models as well as data from the skin of AD patients to understand how filaggrin insufficiency alters the way keratinocytes communicate with the adaptive immune system in the disease, with a focus on CD1a-restricted lipid-specific T-cell responses.

## Materials and methods

### Samples

Ethical approval for the study was obtained from the Independent Bioethics Committee for Scientific Research at the Medical University of Gdańsk (ethical approval numbers: NKBBN/558/2017-2018 and NKBBN/621-574/2020). Buffy coats were obtained from blood donations from healthy donors at the Regional Blood Center in Gdansk.

### Cell culture and media

shC and shFLG HaCaT keratinocytes established by us previously as a stable line by small harpin interference with the use of a lentiviral system (25, 30) were grown in Dulbecco’s Modified Eagle’s Medium (DMEM-high glucose, Sigma-Aldrich) with 10% FBS (Sigma-Aldrich), 2 mM L-Glutamine (Sigma-Aldrich) and 1% Pen/Strep (Sigma-Aldrich) with selection carried out by puromycin at concentration of 20 µg/ml. K562-CD1a cells (a kind gift from Prof. Branch Moody) were cultured in RPMI-1640 (Sigma-Aldrich) with the addition of 200 µg/ml G418 (Thermo Fisher Scientific), 1% Pen/Strep (Sigma-Aldrich), and 10% heat-inactivated FBS (Sigma-Aldrich) and cultured at 37°C and 5% CO_2_. For EV isolation media containing sEV-depleted FBS, treatments were carried out when the cells reached 80%–90% confluence (with the conditioned media being collected at 100% cell confluence; cell count in a region of 25 × 10^6^ cells per T75 flask). T cell medium was prepared by supplementing RPMI-1640 (Sigma-Aldrich) with 5% human male heat-inactivated AB serum (Sigma-Aldrich), 1% Pen/Strep (Sigma-Aldrich), 10 mM HEPES (Sigma-Aldrich), 2 mM L-glutamine (Sigma-Aldrich), 1% non-essential amino acids (Biowest), 50 µM 2-mercaptoethanol (Sigma-Aldrich), and 10 ng/ml IL-2 (PeproTech).

### Flow cytometry

Cells were washed, stained with fluorophore-conjugated antibodies for 30 min at 4°C, and washed in PBS and fixed in 4% formaldehyde (Sigma-Aldrich). Samples were acquired using Guava easyCyte (Millipore), and data were analyzed using GuavaSoft 3.1.1. Antibodies were purchased from BioLegend: CD14-APC, CD40-FITC, CD80-PE, CD86-PE, CD1a-APC at 1:200 dilution, or BD Biosciences: HLA-DR-PE at 1:200 dilution. The catalogue numbers are listed in **Supplementary Table S6**.

### Western blot

Cells were lysed in RIPA buffer (Cell Signalling Technologies) supplemented with cOmplete™, Mini, EDTA-free Protease Inhibitor Cocktail (Roche), centrifuged for 15 min at 4°C and 13,000×*g*, and the supernatant was collected. The lysates or EV samples were heated in Bolt™ LDS Sample Buffer (Invitrogen) for 10 min at 80°C and run on Bolt™ 4%–12% Bis-Tris Plus Gels (Invitrogen) in a Mini Gel Tank (Life Technologies) connected to a PowerEase™ 300 W Power Supply (Life Technologies). The proteins were transferred onto nitrocellulose membranes (iBlot™ 2 Transfer stack; iBlot 2 Dry Blotting System, Invitrogen), and the membranes were blocked with 5% fat-removed milk in PBS. Primary antibody incubations were carried out at 4°C on a shaker overnight, and secondary antibody IRDye^®^ 800CW or IRDye^®^ 680RD (LI-COR Biosciences, Lincoln, NE, USA) (dilution 1:25,000 in PBS with 0.05% Tween 20) for 30 min at RT. The catalog numbers of the antibodies are listed in **Supplementary Table S6**. The membranes were scanned and analyzed using an Odyssey Clx Imaging System (LI-COR Biosciences).

### mRNA microarray

shC and shFLG cells were left untreated or treated with IL-4/IL-13 combination or IFNγ (all cytokines from Peprotech, treatments at 50 ng/ml). After 24 h, RNA was extracted using the RNeasy Plus kit (Qiagen) according to the manufacturer’s instructions, and microarray analysis was performed using Service XS (Holland) on an HT12 BeadArray platform (Illumina). The data were normalized using lumi (31) and analyzed using LIMMA (32). The data were submitted to the Gene Expression Omnibus (GSE203409).

### Monocyte-derived dendritic cell generation and sEV treatment

CD14+ cells were isolated magnetically from PBMCs using the MojoSort™ Human CD14 Selection Kit (BioLegend), according to the manufacturer’s protocol. The cells were grown in 24-well plates (Corning) in RPMI-1640 medium (Sigma-Aldrich) supplemented with 1% Pen/Strep (Sigma-Aldrich), 10% heat-inactivated FBS (Sigma-Aldrich) (complete RPMI), 50 ng/ml GM-CSF, and 1,000 U/ml IL-4 (PeproTech). On days 2 and 4 of culture, the medium was replaced with fresh complete RPMI and cytokines, and the cells were harvested on day 7. To generate mature monocyte-derived dendritic cells (moDCs), LPS (Sigma-Aldrich) was added at 1 μg/ml on day 6. moDCs were incubated with 10 µg/ml of sEVs measured by protein concentration with NanoDrop 2000 (Thermo Fisher Scientific) overnight, and their marker expression was analyzed by flow cytometry.

### EV isolation, purification, and characterization

sEV-free media was used throughout, and the protocol for sEV isolation was as follows: conditioned medium (CM) after 72 h of culture was harvested and centrifuged at 300×g (Megafuge 16R TX-400 centrifuge, Thermo Fisher Scientific) for 10 min to remove cells and cell debris, followed by centrifugation at 2,000×g (Megafuge 16R TX-400 centrifuge, Thermo Scientific) for 10 min to remove insoluble proteins and apoptotic bodies (AP). The supernatant was ultracentrifuged (OptimaTM L-90K or OptimaTM LE-80K ultracentrifuge, Beckman Coulter) at 10,000×*g* (AVG) for 30 min to isolate microvesicles (MVs) and the supernatant was ultracentrifuged at 100,000×*g* (AVG) for 16 h to pellet the exosome-enriched (100 K) fraction. If further purification was required, the exosome-enriched pellet was layered on an iodixanol/sucrose discontinuous gradient (iodixanol concentration ranging between 6% and 18%, increments of 1.2%, 1 ml each fraction). The pellet was top-loaded and ultracentrifuged (OptimaTM L-90K or OptimaTM LE-80K ultracentrifuge; Beckman Coulter) at 198,000×*g* for 2.5 h (SW 41 Ti rotor; Beckman Coulter). Fractions were collected separately (1 ml) and pooled when required, followed by washing with PBS. The top-loaded sample was pooled with the first fraction and fraction 1 (6%+ sample). The sEVs were stored in PBS at −20°C. Quantification and size measurement of sEVs were performed by Nanoparticle Tracking Analysis (NTA) using a NanoSight NS300 equipped with a 488 nm laser (Malvern Instruments). 3 × 30 s recordings was taken for each sample. Electron microscopy was carried out as a service by the Laboratory of Electron Microscopy on Formvar/Carbon film on Copper 300 mesh (EM Resolutions), and samples were imaged using a Tecnai G2 Spirit BioTWIN (FEI Inc.) transmission electron microscope.

### ELISpot and T-cell culture

The Human IFN-γ ELISpot BASIC kit (ALP) (Mabtech) was used to assess T-cell responses. T cells were magnetically selected using the MojoSort™ Human CD3 T Cell Isolation Kit (BioLegend), according to the manufacturer’s protocol, and rested in complete RPMI overnight. Immature moDCs were harvested, washed, and pulsed with sEVs isolated from 1 or 2 mln shC or shFLG cells together with CEFT Pool (JPT Peptide Technologies) at 1 μg/ml per peptide or 10 µg/ml of CMV pp65 protein (ProSpec-Tany TechnoGene Ltd.) overnight. For CD1a-dependent T-cell responses, K562-CD1a cells were pulsed with 1 µg/ml PLA2 (Sigma-Aldrich) and sEVs isolated from 1 or 2 mln shC or shFLG keratinocytes per 50,000 K562-CD1a cells overnight; alternatively, K562-CD1a cells were incubated with equivalent amounts of PLA2-digested sEVs. For single lipid ELISpot, K562-CD1a cells were pulsed with 10 µM myristic acid (C14:0; Sigma-Aldrich), docosahexaenoic acid (C22:6; Sigma-Aldrich), or Lyso-PC18:0 (Cayman Chemical Company). Lipid stocks were prepared as follows: C14:0 at 200 mM in DMSO, Lyso-PC18:0 at 50 mM ethanol with heating at 47°C for 5 min while C22:6 was purchased in liquid form and stored at −20°C and protected from light. The working concentrations of lipids were obtained by dilution in the cell culture medium. Cells were seeded on a pre-coated plate (20,000 immature moDCs or 25,000 K562-CD1a cells per 100,000 T cells) and incubated overnight at 37°C and 5% CO_2_. After ELISpot, cells were harvested and cultured for 13 days in T cell medium with media change every 2–3 days. Then, the cells were rested in complete RPMI and incubated on an ELISpot plate with K562-CD1a cells pulsed with single lipids, as described above. Unstimulated T cells were used as the negative control, and 150 ng/ml PMA (Sigma-Aldrich) and 75 ng/ml ionomycin (Sigma-Aldrich) were added to T cells as the positive control. After overnight incubation, the supernatants were harvested and stored at −80°C for downstream assays. The plate was developed using the AP Conjugate Substrate Kit (Bio-Rad) according to the manufacturer’s protocol and read using Mabtech IRIS™ reader (Mabtech) or AID reader (Autimmun Diagnostika GmbH).

### ELISA

IL-10 levels in cell culture supernatants were measured using the ELISA MAX™ Standard Set Human IL-10 (BioLegend) or Human IL-10 ELISA Set (Diaclone), according to the manufacturer’s instructions, using ELISA Coating Buffer (BioLegend). IL-13 in cell culture supernatants was measured using the Human IL-13 ELISA Development Kit (HRP) (Mabtech) or Human IL-13 DuoSet ELISA (R&D Systems) according to the manufacturer’s instructions. For IL-17A measurement Human IL-17A ELISA Development Kit (HRP) (Mabtech) was used, according to the manufacturer’s instructions. Nunc-Immuno™ MicroWell™ 96 well plates (Sigma-Aldrich) were used. Plates were developed using the TMB Substrate Set (BioLegend), with H_2_SO_4_ added to stop the reaction. Absorbance was read at 450 nm and 570 nm wavelengths using an Epoch 2 Microplate Spectrophotometer (BioTek) or an Asys UVM340 microplate spectrophotometer (Biochrom). The absorbance at 570 nm was subtracted from that at 450 nm, and the concentrations were calculated based on standard curve equations.

### cPLA activity and PLA2 cell-free digestion

Calcium-dependent cytosolic phospholipase A2 (cPLA2) content in cell lysates and sEVs was assessed by measuring the activity of the enzyme towards a synthetic substrate, arachidonoyl thio-PC, with the cPLA2 Assay Kit (Cayman Chemical), according to the manufacturer’s instructions. Supernatants obtained after centrifugation (14,000×*g*, 10 min, 4°C) of lysed samples were tested in duplicate, and the reaction mixture was incubated for 5 min and then overnight. Absorbance was measured at 414 nm and 405 nm. For the cell-free digestion of cPLA Assay Buffer, a component of the cPLA2 Assay Kit (Cayman Chemical) was diluted in PBS according to the manufacturer’s protocol; Ca^2+^ concentration was adjusted to 20 mM with CaCl_2_. sEVs and 1 μg/ml active or heat-inactivated (95°C, 15 min) PLA2 (Sigma-Aldrich) were then added. After 1 h of incubation, the samples were stored at −20°C.

### Protein mass spectrometry

sEVs isolated from an equal number of cells in equal volumes of conditioned media were used as the starting samples for the analysis, with equal sample loading volumes per analysis. Cells and sEVs were lysed in lysis solution containing 1% SDS, 100 mM Tris–HCl pH 8.0, 50 mM dithiothreitol, and incubated at 95°C for 10 min before the sEVs were purified by gradient to remove contaminating protein aggregates, including keratohyalin granules, potentially found in the conditioned media separately to EVs if released from dying cells. Protein concentration was measured using a spectrophotometer (measurement at 280 nm), and equal amounts (100 µg) of each sample were transferred to separate 10 kDa Microcon filters (Merck-Millipore, Burlington, MA, USA). Samples were prepared for mass spectrometry analysis in a Filter Aided Sample Preparation (FASP) procedure (33), including washes with 8M urea in 100 mM Tris-HCl, pH 8.5 (by centrifugation at 10,000×*g*), followed by cysteine alkylation using 55 mM iodoacetamide in urea. Proteolytic digestion by trypsin added to the samples in a 1:50 weight ratio was carried out at 37°C overnight; 100 μg of protein per sample was used in this step. Equal amounts of the obtained digests (10 μg) were desalted using the STAGE Tips (34) procedure onC18 resin. LC-MS/MS analysis was conducted with 1.67 ug of the peptides per sample,and the measurements were conducted on a Triple TOF 5600+ mass spectrometer (SCIEX, Framingham, MA) operating in positive ion mode coupled with an Ekspert MicroLC 200 Plus System (Eksigent, Redwood City, CA). Liquid chromatography separation was performed on a ChromXP C18CL column (3 µm, 120 Å, 150 × 0.3 mm) in a gradient of 11%–42.5%B for 60 min (buffer A: 0.1% formic acid in water, buffer B: 0.1% formic acid in acetonitrile) at a flow rate of 5 µL/min. The instrument was operated using the Analyst TF 1.7.1 software (SCIEX Framingham, MA). The precursor ions were fragmented via collision-induced dissociation (CID). Data dependent acquisition (DDA) runs consisted of a TOF scan in the m/z range of 400 Da–1,200 Da in 100 ms and a subsequent Product Ion scan in the m/z range of 100 Da–1,800 Da in 50 ms, resulting in the cycle time of 1.15 s. All samples were measured in the data-dependent acquisition mode for spectral library construction and by Sequential Window Acquisition of all Theoretical Mass Spectra SWATH-MS (35) method in triplicate for relative quantification. Separate spectral libraries for the cell and sEV samples were built by database search carried out in ProteinPilot 4.5 software (SCIEX) against a SwissProt Homo sapiens database (version from 7 February 2020); only proteins identified at 1% FDR were considered valid identifications SWATH-MS measurements were processed with respective libraries in the PeakView 2.2. software. The resulting protein intensities were normalized by total area sums (TAS), i.e., to normalize to the total amount of detected peptide approach, and imported into the Perseus software (36), where the technical replicates were median-averaged, and the resulting values were log2-transformed and normalized by z-score. A t-test between the test and control groups was conducted, and the results with FDR-adjusted p-values lower than 0.05 were statistically significant. The mass spectrometry proteomics data were deposited in the ProteomeXchange Consortium via the PRIDE partner repository with the dataset identifier PXD026859 (37).

### Lipid mass spectrometry

sEVs isolated from the equal number of cells in equal volumes of conditioned media were the starting samples for the analysis; with equal sample loading volumes run per analysis. The samples were extracted in a cold chloroform/methanol mixture (1/2, v/v), followed by the addition of chloroform and deionized water to separate the aqueous and organic phases. The bottom layer derived from the cell extract was used directly in the LC–MS analysis; the sEV lipid extract was dried and dissolved in methanol. The acquisition was performed on an Agilent 1290 LC system coupled to a 6540 Q-TOF–MS (Jet Stream Technology, Agilent Technologies). Lipid separation was achieved using a reversed-phase column (Poroshell 120 EC-C8; Agilent InfinityLab; Agilent Technologies) maintained at 60°C. The two most abundant peaks were then selected for fragmentation. Lipidomic data were processed on the Agilent MassHunter Workstation Profinder 10.0 (Agilent Technologies) using the Molecular Feature Extraction (MFE) algorithm, followed by Targeted Molecular Feature Extraction, and data alignment and filtration were carried out using Mass Profiler Professional 15.1 software (Agilent Technologies); missing values were exported as missing data. Filtration was based on the frequency (the MFs remained in the dataset if they were present in 80% of the samples in at least one specified group) and QC %RSD. The MFs present in the extraction blank with an average peak volume higher than 10% of the average peak volume in the real samples were removed. Further statistical analysis was conducted using MetaboAnalyst5.0 (https://www.metaboanalyst.ca/home.xhtml), reporting an adjusted p-value threshold <0.05 (unpaired t-test, unequal variance, Benjamini–Hochberg FDR correction). Missing values were replaced with 1/5 of the minimum positive value of each variable if not detected in only one sample group, or by the mean peak area of the compound in a group of samples if not detected in only one of four biological replicates or incorrectly integrated by the software. The levels of individual lipid species were normalized to the total amount of corresponding lipid classes. The peak area of a particular lipid species was divided by the sum of the peak areas of the lipids of a specific class to ensure that the amount of starting material did not bias the comparative analysis results. Euclidean distance and Ward clustering algorithms were used for the heatmap. The data (relative amounts of lipids within a class) were log-transformed (base10) for a heatmap, and log-transformed and autoscaled (mean-centered and divided by the standard deviation of each variable) for PLS-DA analysis. Lipid identification was carried out by searching the custom lipid database of theoretical lipid structures based on an accurately measured m/z value (Δ5 ppm tolerance), followed by manual interpretation of the obtained MS/MS spectra.

### Functional enrichment and Gene Ontology analysis

**Figure 1:**
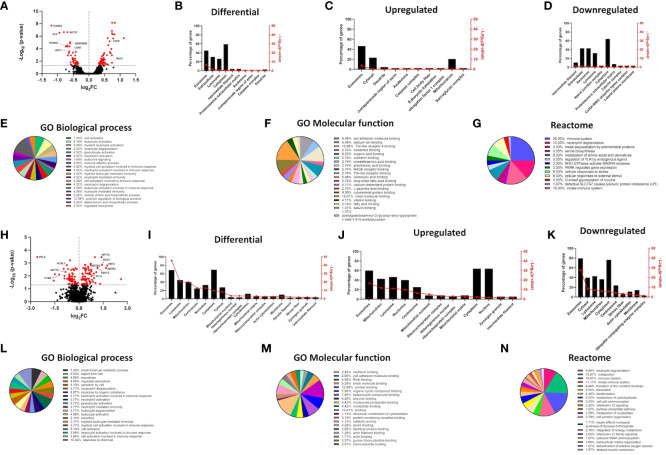
mRNA and protein expression signatures signify alterations in GO terms for proteins enriched in the exosomal/EV compartment in filaggrin-insufficient keratinocytes. **(A)** Volcano plot depicting mRNA expression changes in shFLG keratinocytes; n = three biological replicates; moderated t-test; FC values were log_2_-transformed and p-values were log_10_-transformed; differentially expressed genes with p <0.05 in red; symbols of selected epidermal barrier- and immune response-related genes are shown; **(B–D)** FunRich analysis showing differential expression of genes encoding proteins enriched within cellular compartments; **(B)** total, **(C)** upregulated and **(D)** downregulated in shFLG; **(E–G)** Gene Ontology and Reactome terms related to genes encoding proteins identified in sEVs by FunRich, differentially expressed in shFLG keratinocytes; analysis by Panther tool; enrichment in GO terms related to: **(E)** biological process, **(F)** molecular function and **(G)** Reactome terms; **(H)** Volcano plot depicting protein expression changes in shFLG keratinocyte cultures; n = four biological replicates; Benjamini-Hochberg FDR; FC values were log_2_-transformed and p-values were log_10_-transformed; differentially expressed proteins with p <0.05 in red; symbols of selected epidermal barrier-, lipid metabolism-, and immune response-related proteins are shown; **(I–K)** FunRich analysis showing differential expression of proteins enriched within cellular compartments; **(I)** total, **(J)** upregulated, and **(K)** downregulated in shFLG; **(L–N)** Gene Ontology and Reactome terms related to proteins identified by FunRich in sEVs, differentially expressed in shFLG keratinocytes; analysis by Panther tool; enrichment in GO terms related to **(L)** biological process, **(M)** molecular function and **(N)** Reactome terms; FC, fold change.

**Figure 2:**
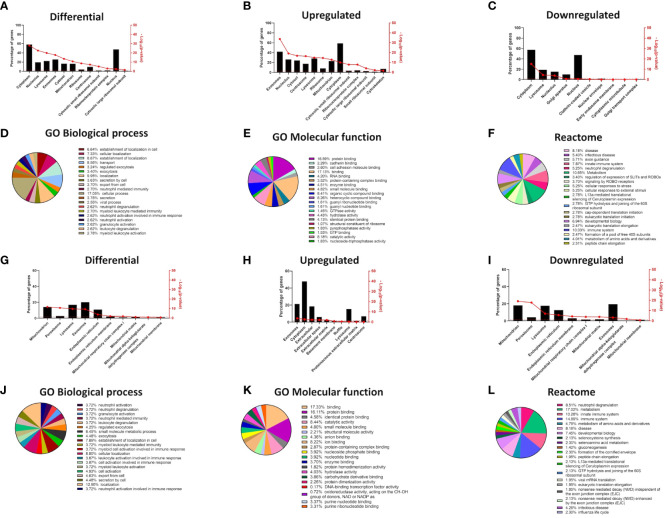
Changes in cellular compartments are signified by differential GO term enrichment in the epidermal organotypic models and skin of atopic dermatitis patients. **(A–C)** Differential expression of proteins enriched in cellular compartments of siFLG organotypic cultures by FunRich tool; **(A)** total, **(B)** upregulated, and **(C)** downregulated in siFLG; **(D–F)** Gene Ontology and Reactome terms related to the proteins identified by FunRich in sEVs, differentially expressed in siFLG organotypic cultures; analysis by Panther tool; enrichment in GO terms related to **(D)** biological process, **(E)** molecular function, and **(F)** Reactome terms. **(G–I)** FunRich analysis showing enrichment of differentially expressed genes encoding proteins within cellular compartments in AD skin; **(G)** total, **(H)** upregulated, and **(I)** downregulated in AD skin; **(J–L)** Gene Ontology and Reactome terms related to the FunRich-identified proteins enriched in sEVs, encoded by genes differentially expressed in AD skin; analysis by Panther tool; enrichment in GO terms related to **(J)** biological process, **(K)** molecular function, and **(L)** Reactome terms.

Cellular compartment enrichment analysis of the omics datasets was performed using FunRich 3.1.3 software. The Vesiclepedia (38) database, available within the software, was used to investigate the association of proteins/gene products identified in omics studies with sEVs. Gene ontology (GO) and Reactome pathways analysis were carried out via the Gene Ontology tool, available at http://geneontology.org/. The complete GO annotation datasets were used in this study. For GO analysis in **Figures 1**, **2** the top 20 GO terms and Reactome pathways for every dataset were selected based on the lowest FDR values, and the number of genes identified within every top 20 term was added together for subsequent pie chart analysis. At the time of analysis, the latest update for GO database was on 01-02-2021 and Reactome database on 17-11-2020.

High-quality data generated in a thoroughly described and validated 3D organotypic model, as well as the mRNA data obtained from the pediatric AD cohort by the Brown lab (23, 24), were re-analyzed to provide new information. We similarly re-analyzed our previously generated shC and shFLG cells mRNA microarray dataset deposited to the Gene Expression Omnibus (GEO) repository and available at the accession number: GSE203409 as well as the proteomics dataset generated within this study as specified in the proteomics section above, which is deposited to the ProteomeXchange Consortium via the PRIDE partner repository with the dataset identifier PXD026859 (37).

### Statistical analysis

One-way analysis of variance (ANOVA) tests with the indicated correction methods were performed using GraphPad Prism v.7.04 or newer (GraphPad Software). Error bars represent SEM, as indicated.

## Results

### Filaggrin insufficiency in keratinocytes affects GO terms related to the sEV compartment

Apart from the widespread disturbances of the structural components resulting in the cardinal features of the AD epidermis, isolated filaggrin insufficiency in keratinocytes also affects additional, seemingly unrelated functions in these cells. Here, we hypothesized that the sEV compartment was also disturbed, influencing the message conveyed between keratinocytes and immune cells in the disease context. While primary keratinocytes growing in a monolayer are rather inefficient sEV producers and obtaining enough of those from keratinocytes isolated from atopic skin punch biopsy or 3D organotypic model is not feasible, the epidermis comprises 10–15 layers of live keratinocytes, which together produce many sEVs. Hence, the potential impact of filaggrin insufficiency is likely to be substantial on the skin. Here, to overcome the issue of low sEV production from the primary cell monolayer and still be able to conclude on the mechanisms relevant to AD, a filaggrin knock-down keratinocyte line that we previously established by shRNA interference (21, 25, 30) was the model of choice. We found extensive changes in the mRNA expression pattern between shC and shFLG cells, with a pronounced difference in expression of keratinocyte-specific genes (**Figure 1A**) and we further proceeded with data analysis using the FunRich tool (39). The advantage of using this tool is that apart from the standard gene ontology (GO) terms for compartmental localization, it also integrates datasets available within Vesiclepedia (38), i.e., a database of proteins specifically enriched within extracellular vesicles (with the term “exosomes” being used for sEVs). This provides better insights into the changes relevant to the sEV compartment, and indeed, the analysis determined that among the many compartments affected, the sEV compartment was most significantly altered (**Figures 1B–D**). More than 40% of the differentially regulated genes encode proteins known to be associated with sEVs. Interestingly, the change was most pronounced for the upregulated genes and less pronounced for the downregulated genes. The dataset filtered for enrichment within this compartment was subsequently used for the enrichment analysis of GO terms for biological processes and molecular functions, as well as Reactome pathways (by Panther tool (40) and Reactome Knowledgebase (41), respectively). This identified extensive differences between filaggrin-sufficient and -insufficient cells at the mRNA level, showing changes in several biological processes related to immune cell activation, molecular function of cell adhesion and molecule binding, and pathways for immune cell activation (predominantly in innate immunity) and stress response (**Figures 1E–G**, **Supplementary Table S1**). Next, we compared shC and shFLG cells at the protein level using mass spectrometry (**Figure 1H**). This yielded similar FunRich outcomes, although we also identified significant downregulation of exosome-relevant proteins (**Figures 1I–K**). GO term enrichment findings aligned with those from the mRNA data (**Figures 1L–N**, **Supplementary Table S2**) with respect to immune activation and cell adhesion, and processes related to exocytosis, secretion, and cellular export were the most prominent in this dataset.

Our 2D monolayer model overcomes the limitation of insufficient sEV yields compared to 3D systems or skin samples, but the drawback is the potential lack of stratification-specific effects. Hence, to ensure that the differential outcome we observed was also relevant at the level of complex epidermal tissues, we also analyzed extensive proteomic data from the filaggrin-insufficient organotypic epidermal model published by Elias et al. (24) (**Figures 2A–F**, **Supplementary Table S3**), as well as the transcriptome dataset obtained from the skin samples of patients with AD (42) (**Figures 2G–L**, **Supplementary Table S4**). The results of the analysis were in strong agreement with those obtained for the monolayer experiments, confirming that filaggrin insufficiency leads to significant alterations within the sEV compartment in 3D tissues among other cellular compartments (lysosomes, cytosol/cytoplasm, nucleolus, and mitochondria; **Figures 2G–I**). Despite some differences likely consequential to the complexity of the stratified epidermis, GO terms related to the biological processes of exocytosis/cellular export and immune cell activation were also enriched in both datasets. Binding and cellular adhesion were clearly identifiable, and pathways related to the immune system, cellular metabolism, and stress response were prominent (**Figures 2J–L**, **Supplementary Table S4**). These results provide cross-validation and increased confidence in the cellular model.

As for the GO terms related to antigen presentation, only the analysis of the *FLG* knockdown organotypic skin model revealed relevant terms; specifically, the terms associated with general as well as MHC class I-specific antigen processing and presentation were enriched, and similar terms were identified by the Reactome pathways (**Supplementary Figure S1**).

### sEVs secreted by filaggrin sufficient and insufficient keratinocytes display similar size and marker characteristics

**Figure 3:**
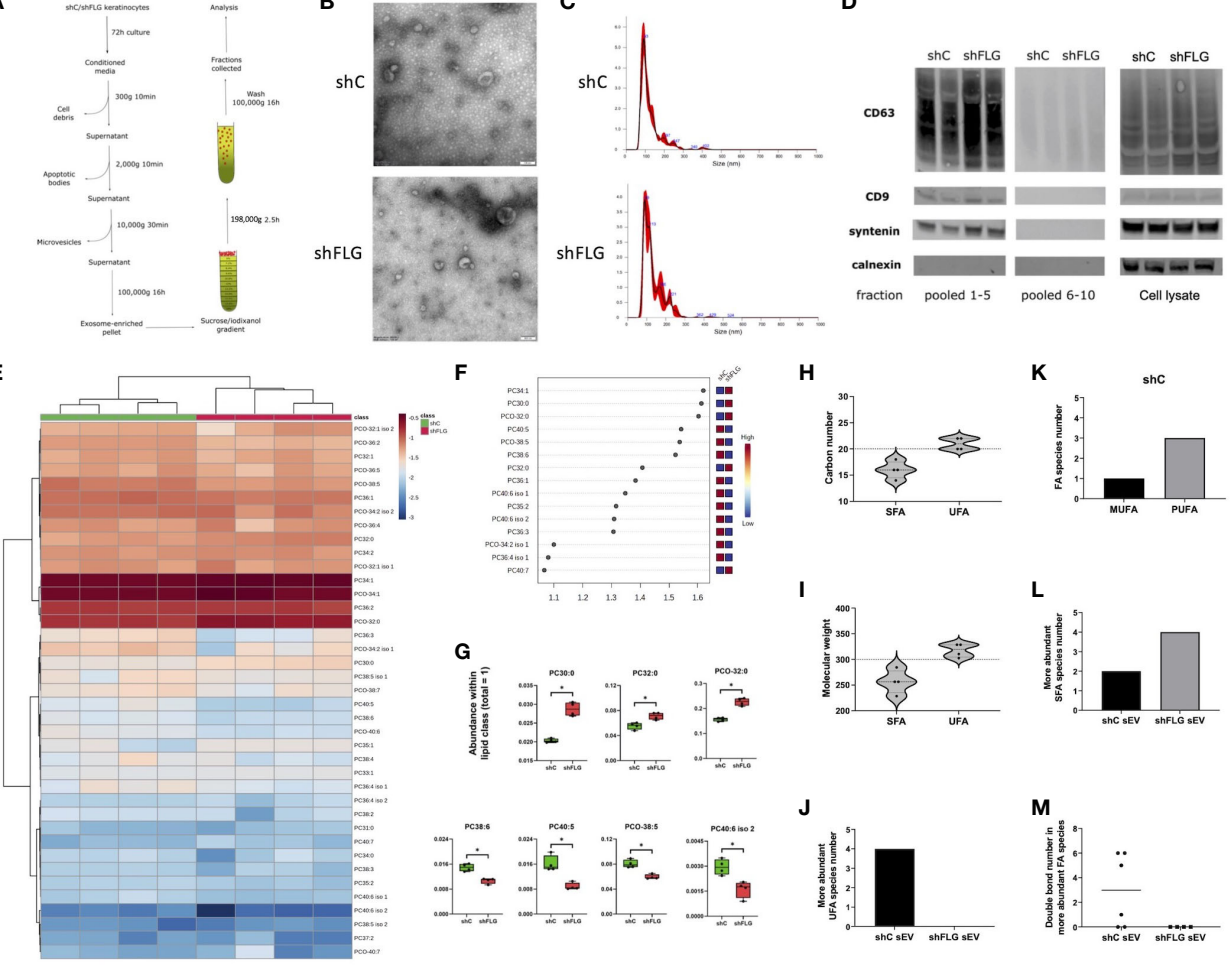
Filaggrin insufficiency alters the sEV composition of PLA2-digestible lipids. **(A)** A protocol for isolation of extracellular vesicles by ultracentrifugation; exosome-enriched sEVs are pelleted as 100K fraction and purified by density gradient; **(B)** Electron microscopy pictures of sEVs preparations; representative of n = 3; **(C)** Size distribution of purified sEVs by Nanoparticle Tracking Analysis (NTA); representative example shown; **(D)** Enrichment of exosomal markers in purified sEVs; Western blot; representative blot, n = 2; pooled fractions 1–5 are purified exosome-enriched sEV; pooled fractions 6–10 are smaller microvesicles; **(E–G)** Lipidomic analysis of PLA2-digestible lipid species in sEVs; **(E)** heatmap of the detected lipids; **(F)** lipid species most affected by filaggrin accordingly to the PLS-DA analysis; the variance importance for the projection values (VIP) were used to sort lipids accordingly to their contribution to PLS-DA model; **(G)** boxplots showing lipid species significantly different in abundance; combined data from n = four biological replicates; unpaired t-test, FDR correction; *, p <0.05; **(H, I)** Fatty acid composition of sEV phospholipids differing in abundance by **(H)** a chain length or **(I)** molecular weight; dotted line shows the length and mass benchmarks for highest CD1a-dependent response; **(J)** Number of the more abundant UFA species in sEVs; **(K)** Breakdown of UFA species from **(J)** in shC_sEV_ by degree of unsaturation; **(L)** Number of the more abundant SFAs in sEVs; **(M)** Number of double bonds in the more abundant FA species in sEVs; PLA2, phospholipase A2; VIP, variable importance in projection; FA, fatty acid; UFA, unsaturated fatty acid, SFA, saturated fatty acid; MUFA, monounsaturated fatty acid; PUFA, polyunsaturated fatty acid; PC, diacylglycerophosphocholine; PCO, ether-linked glycerophosphocholine.

Next, we next isolated exosome-containing sEV fractions from conditioned keratinocyte media using an ultracentrifugation protocol (**Figure 3A**). Vesicles were examined using electron microscopy and Nanoparticle Tracking Analysis (NTA), and we confirmed the characteristic cup shape and size distribution (**Figures 3B, C**), demonstrating exosome enrichment. We did not observe any substantial differences with respect to vesicle sizes or secretion levels between shC and shFLG cells (**Supplementary Figure S2**). The 100 K pellets fractionated on a sucrose/iodixanol gradient contained high levels of exosomal markers CD9, CD63, and syntenin-1 in the top fractions (fractions 1–5; **Figure 3D**), but not in the lower fractions (fractions 6–10), suggesting no significant contamination by small microvesicles (MVs), which displayed lower but still detectable CD9/CD63 levels (43, 44), indicating that the 100 K pellet contained a relatively pure exosomal population. However, we did not find any substantial differences in the expression of the markers between the sEVs obtained from shFLG cells (shFLG_sEV_) and those from shC cells (shC_sEV_).

### sEVs secreted by filaggrin insufficient keratinocytes do not impact responses to peptides or whole proteins

Subsequently, we determined the capacity of shC_sEV_ and shFLG_sEV_ to affect antigen presentation to T cells. To this end, we first tested the effect of the vesicles on dendritic cells but did not observe any differential outcomes regarding the expressed surface markers in either immature or mature monocyte-derived dendritic cells (moDCs) (**Supplementary Figures S3A, B**). Next, we compared the effects of shC_sEV_ and shFLG_sEV_ on specific T cell IFNy responses to peptide antigens. We used a panel of MHC class I- and class II-restricted peptides derived from common pathogens and vaccination strains, to which most of the population has been exposed in their lifetime and maintains memory responses (CEFT peptide pool). Hence, in subsequent experiments, we tested whether the addition of sEVs to immature moDCs during the antigen pulsation period with CEFT affects the downstream T cell response to this peptide pool. The ELISpot assay experiments did not reveal any major differences in the IFNy response level between cells stimulated with CEFT in combination with shFLG_sEV_ and shC_sEV_ (**Supplementary Figure S3C**) in comparison to the pulsation with the CEFT peptides alone. We also tested IFNy T cell responses induced by a whole protein, using CMV pp65, to which most of the population has immunological memory, provided as an antigen source (**Supplementary Figure S3D**). Here, we obtained similar results, i.e., no significant differences in comparison to the pp65-only control.

### sEVs secreted by filaggrin insufficient keratinocytes display altered lipid profile

While we did not observe any differential outcomes from MHC class I/class II-restricted T cells, we considered it plausible that lipid presentation could be affected. Given that the skin is a body site highly dependent on CD1a-mediated T cell responses and CD1a^+^ cells are abundant, we next assessed the effect of sEV on CD1a-mediated lipid-specific responses. We previously determined the role of phospholipase PLA2 in neoantigen generation and induction of T cell reactivity via this pathway (4, 5). We also showed that mast cell-derived sEVs may contain active PLA2 enzyme and supply it to induce neoantigen-specific T-cell responses (10). Hence, we investigated whether keratinocytes express considerable amounts of the enzyme that can be enclosed within sEVs. However, our mass spectrometry data for the cell lysates suggested this was not the case (**Supplementary Table S5**); similarly, we did not detect any relevant enzymatic activity in either the keratinocyte lysates or sEVs when testing for the PLA2 activity which detects both the secretory and cytosolic PLA2s (**Supplementary Figure S4A**). This ruled out the possibility that PLA2 may be supplied to sEVs secreted by keratinocytes in the steady state and that filaggrin is insufficient. However, because sEVs are lipid-based organelles, they could potentially provide a source of lipid ligands to CD1a-restricted T cells. Hence, we followed the mass spectrometry lipidomic profiling of shFLG_sEV_ and shC_sEV_, confirming that the sEV lipid content was biased towards phospholipids, as expected. In terms of changes in the relative content between shFLG_sEV_ and shC_sEV_, we found substantial alterations among PLA2-digestible lipid classes, specifically diacyl glycerophosphocholines (PCs) and ether analogs (PCOs) (**Figures 3E–G**, **Supplementary Figure S4B**).

### Filaggrin insufficiency background narrows the repertoire of sEV-derived lipids most suitable for CD1a binding

As far as the CD1a-mediated presentation is concerned, the size and topology of the CD1a binding groove defines the suitability of lipids of various lengths and structural complexity to bind and form stable complexes with the molecule. To this end, Nicolai et al. (45) elegantly documented that ligands of around 20 carbon atoms and a molecular weight of approximately 300 are optimal; most ligands promoting strong T-cell activation fell within those ranges. Similarly, features of added structural complexity, such as the presence of unsaturated bonds, also improve CD1a-restricted T cell responses in comparison to fully saturated chains (45). We observed an increased contribution of saturated or monounsaturated long-chain fatty acid (LCFA)-containing PCs and decreased content of very long chain polyunsaturated fatty acid (PUFA)-containing PCs (e.g., C22:6; docosahexaenoic acid; DHA, in PC40:6 and PC38:6) in shFLG_sEV_ vs shC_sEV_ (**Figure 3G**). Hence, we next assessed the breadth of the potential antigenic lipid repertoire within the sEV compartment, considering phospholipid fatty acid constituents. Unsaturated fatty acids (UFAs; both mono- and polyunsaturated FAs; MUFAs and PUFAs) detected in sEVs closely matched the optimal length and size in terms of carbon number (**Figure 3H**) and molecular weight (**Figure 3I**) benchmarks in comparison to saturated fatty acids (SFAs). This suggests that UFAs were more likely to affect CD1a-mediated responses. When lipid sources were compared, it was clear that FAs identified as more abundant in shC_sEV_ represented a much greater variety and were more suitable for CD1a presentation than those in shFLG_sEV_ (**Figure 3J**). Strikingly, when assessing the saturation of the FA chains, we found no single UFA to be more abundant in the shFLG_sEV_ (**Figure 3J**); at the same time, we detected three times more PUFA than MUFA species in shC_sEV_ (**Figure 3K**). In contrast, the SFA content showed the reverse trend, i.e., we found a much greater number of SFAs within the pool of more abundant FAs in shFLG_sEV_ (**Figure 3L**). Lastly, the number of double bonds in the FA chains also differed greatly, with no single FA being more abundant in shFLG_sEV_ (**Figure 3M**).

### sEVs secreted by filaggrin insufficient keratinocytes modulate CD1a-autoreactive T cell responses

**Figure 4:**
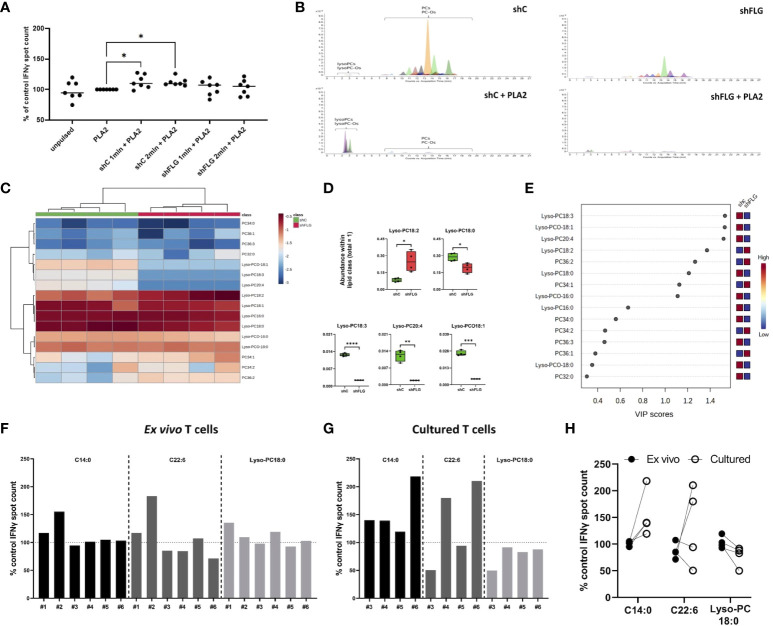
shFLG_sEV_ demonstrate a reduced capacity to stimulate CD1a-specific T-cell responses. **(A)** IFNy responses of T cells stimulated with K562-CD1a cells pulsed with 1 µg/ml PLA2 and sEVs from 1 or 2 million keratinocytes overnight measured by ELISpot assay; means ± SEM shown; data normalized to control = 100%; n = seven donors; one-way ANOVA with Šídák’s multiple comparisons test; **(B)** Extracted Ion Chromatograms (EICs) showing sEV lipid profile before and after digestion with 1 µg/ml PLA2 for 1 h (n = 4; representative data shown); **(C–E)** Lipidomic analysis of glycerophosphocholine-related products after sEV digestion; **(C)** heatmap of detected lipids; **(D)** boxplots showing lipid species significantly different in abundance; data from n = four biological replicates, unpaired t-test, FDR correction; **(E)** lipid species most affected by filaggrin insufficiency accordingly to the PLS-DA analysis; the variance importance for the projection values (VIP) were used to sort lipids accordingly to their contribution to PLS-DA model; **(F–H)** IFNy responses from **(F)** *ex vivo* T cells stimulated with K562-CD1a cells pulsed with 10 µM of lipids overnight; n = six donors; and **(G)** T cells cultured for 13 days following ELISpot, n = four donors; means from two technical replicates for each individual donor, normalized to the control=100% are shown; **(H)** comparison of responses between *ex vivo* and cultured T cells from n = four donors represented both in **(F, G)**; one-way ANOVA with Šídák’s multiple comparisons test. PLA2, phospholipase A2; VIP, variable importance in projection. PC, diacylglycerophosphocholine; Lyso-PC, monoacylglycerophosphocholine; Lyso-PCO, monoalkylglycerophosphocholine; C14:0, tetradecanoic acid; C22:6, docosahexaenoic acid; *, p <0.05; **, p <0.01; ***, p <0.001; ****, p <0.0001.

To determine if the differential content of CD1a ligands in intact sEVs translates into differences in T cell reactivity we next proceeded with the IFNγ ELISpot assay. Here, we used a CD1a transfected K562 cell line, devoid of class I and II expression, as antigen-presenting cells (K562-CD1a; **Supplementary Figure S4C**), which has been successfully used in several studies investigating CD1a-mediated T cell responses (1, 4, 5, 8, 45–47). While we noted some reactivity with certain donors manifesting a level of IFNγ production, this was not significant in comparison to the unpulsed control cells or between the cellular sEV sources, regardless of the filaggrin status (**Supplementary Figure S4D**). Next, with the aim of liberating lipids from the sEV membranes, we followed with the addition of bee venom PLA2 as a source of the enzymatic activity to generate lipid neoantigens; pulsing of the cells with sEVs and PLA2 was carried out simultaneously. Interestingly, we observed that the addition of shC_sEV_, together with PLA2, resulted in the induction in CD1a-specific IFNy responses above the “PLA2 only” level (**Figure 4A**), indicating that digestion of sEVs secreted by filaggrin-sufficient keratinocytes released lipids suitable for CD1a-dependent T cell activation. In contrast, the addition of shFLG_sEV_ failed to induce IFNγ T cell responses above the control level, and we measured IL-10 and IL-13 secretion in the supernatants by ELISA, but the levels produced were negligible in this system (**Supplementary Figure S4E**).

### Filaggrin insufficiency reduces the complexity of sEV lipid composition and diversity of the ligands promoting homeostatic responses

Next, to determine the lipid species that may be involved in the differential outcomes, we subjected sEVs to PLA2 treatment in a cell-free assay. We observed that phospholipids in both shFLG_sEV_ and shC_sEV_ were completely digested by the enzyme and disappeared from both shC_sEV_ and shFLG_sEV_ samples (**Figure 4B**). A low signal for PCs was detected only for the most abundant product species, such as lysoglycerophosphocholines (lysoPCs and ether analogs lyso-PCO), and the relative content of almost all the detected lyso-PCs and lyso-PCOs was much lower in the digested shFLG_sEV_ than in the digested control shC_sEV_ (**Figures 4C–E**, **Supplementary Figure S5A**). Lyso-PC18:0 was the most abundant species within its lipid class found in keratinocyte-derived sEVs and was also significantly decreased in shFLG_sEV_; shFLGsEV were also lower in the content of all differentially abundant Lyso-PCs and Lyso-PCOs apart from Lyso-PC18:2 which showed an opposite trend (**Figure 4D**).

To further define the impact of the lipids contained within the shC_sEV_ on the observed T cell reactivity, we selected three lipids found in sEVs, with representative acyl chain lengths and molecular weights that reflected optimal and suboptimal characteristics for ligand binding to CD1a. Specifically, we included a short-chain SFA (C14:0), a long-chain PUFA (C22:6; DHA), and lysophosphatidylcholine (Lyso-PC18:0) and tested their capacity to promote IFNγ responses in peripheral blood T cells. We observed low but detectable responses to all lipids in some donors *ex vivo* (**Figure 4F**). Culturing the T cells into short-term lines augmented the responses to C14:0 and C22:6, but the responses of the cultured cells to Lyso-PC18:0 were reduced (**Figures 4G, H**).

### sEVs secreted by filaggrin insufficient keratinocytes contain more non-permissive/inhibitory lipids capable of CD1a binding and dampening T cell responses

**Figure 5:**
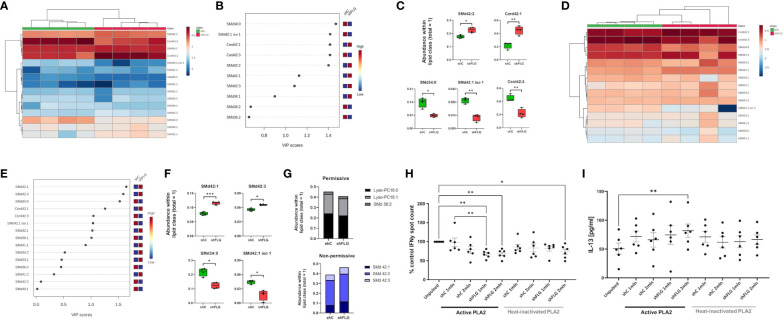
Non-permissive CD1a lipid antigens are enriched in sEVs secreted by filaggrin-insufficient keratinocytes. **(A–C)** Lipidomic analysis of PLA2-non-digestible lipid species in sEVs; **(A)** heatmap of all detected lipids; **(B)** lipid species most affected by filaggrin insufficiency accordingly to the PLS-DA analysis; the variance importance for the projection values (VIP) were used to sort lipids accordingly to their contribution to PLS-DA model; **(C)** boxplots showing lipid species significantly different in abundance; n = four biological replicates, unpaired t-test, FDR correction; **(D–F)** Lipidomic analysis of PLA2-non-digestible lipid species in sEVs digested with 1 µg/ml PLA2 for 1 h; **(D)** heatmap of all detected lipids; **(E)** lipid species most affected by filaggrin insufficiency accordingly to the PLS-DA analysis; the variance importance for the projection values (VIP) were used to sort lipids accordingly to their contribution to PLS-DA model; **(F)** boxplots showing lipid species significantly different in abundance; n = four biological replicates; unpaired t-test, FDR correction; **(G)** Relative amounts of permissive and non-permissive species in PLA2-digested sEVs; **(H)** IFNy responses by T cells stimulated with K562-CD1a cells pulsed overnight with sEVs from 1 or 2 million keratinocytes digested with 1 µg/ml PLA2 for 1 h; n = six donors; data normalized to control = 100%; **(I)** IL-13 secretion into culture supernatants from **(H)** measured by ELISA; n = six donors; means ± SEM are shown; one-way ANOVA with Šídák’s multiple comparisons test; *, p <0.05; **, p <0.01; PLA2, phospholipase A2; VIP, variable importance in projection; SMd, sphingomyelin; Cerd, ceramide.

In addition to phospholipids, which are classical PLA2 substrates, we determined that sEVs contain many lipids that are not preferential targets for PLA2-mediated enzymatic cleavage, such as ceramides and sphingolipids (**Figures 5A–C**, **Supplementary Figure S5B**). Accordingly, we found that the relative proportion within classes of these lipids did not change upon PLA2 digestion, and their relevant shC_sEV_ vs shFLG_sEV_ contribution remained comparable to that in the untreated samples (**Figures 5D-F**). However, while resistant to the digestion process itself, these lipids would also be liberated from sEVs due to the perturbing impact of PLA2 on vesicular membranes and so, would be present in the lipid mixture after digestion and their impact may be important; specifically, a recent study by Cotton et al. identified a propensity of CD1a to preferentially bind endogenous non-permissive lipid ligands that inhibit T cell responses (CD1a blockers) (48). Hence, we attempted to determine whether any of the detected non-digestible lipids had the potential to reduce CD1a reactivity. Indeed, we found that the keratinocyte-derived sEVs contained sphingomyelins, non-permissive ligands capable of strongly binding to CD1a (48), i.e., SMd42:1, SMd42:2, and SMd42:3 (**Supplementary Figure S5D**) which exhibit blocking potential on T cell activation.

To obtain a clearer picture of the relative sEV content of candidate permissive and non-permissive CD1a ligands, we classified the lipid species based on published data (4, 10, 47–49). The results of our analysis showed that shFLG_sEV_ were less abundant in some permissive ligands, i.e., Lyso-PC16:0, Lyso-PC18:1, and SMd36:2 (**Figure 5G**). In contrast, we observed greater enrichment of shFLG_sEV_ in non-permissive ligands, i.e., inhibitory very long-chain sphingomyelins; experimentally tested SMd42:1, SMd42:2 and SMd42:3, predicted as non-permissive because of their structural features (very long chain and protruding headgroup) (**Figure 5G**). Interestingly, we observed an opposite trend for the SMd42:1 isomer, which was slightly more abundant in the shC_sEV_. However, while the structure of this isomer is unknown, the abundance of SMd42:1 species in shFLG_sEV_ has the same composition as that shown to be non-permissive by Cotton et al. (48).

With these new insights, we recognized that the enrichment of lipids with inhibitory function in shFLG_sEV_ could have interfered with the ELISpot assay, reducing the detectable IFNγ T cell response, which might have partly depended on the self-ligands liberated from the membranes of cells exposed to PLA2 at the time of sEV pulsation, which could potentially mask some of the differential effects. Hence, we conducted another assessment of IFNγ responses using sEV already digested by PLA2 and included heat-inactivated PLA2 controls to confirm the active enzyme dependency. We observed that PLA2-digested shFLG_sEV_ significantly inhibited IFNγ secretion from T cells, whereas the addition of digested shC_sEV_ did not result in any differential outcomes (**Figure 5H**). In addition, we also noted a significant decrease in the IFNγ response to the higher shFLG_sEV_ concentration even in the heat-inactivated PLA2, possibly due to the spontaneous release of some inhibitory lipids from these vesicles (**Figure 5H**). In contrast to the IFNγ response, we observed stimulation of IL-13 by shFLG_sEV_ but not by shC_sEV_ and a subtle similar trend in IL-17A production, but no difference in IL-10 levels (**Figure 5I**, **Supplementary Figures S6A, B**).

### Changes in the lipid composition of sEVs secreted by filaggrin insufficient keratinocytes reflect the shift in the cellular lipid landscape

**Figure 6:**
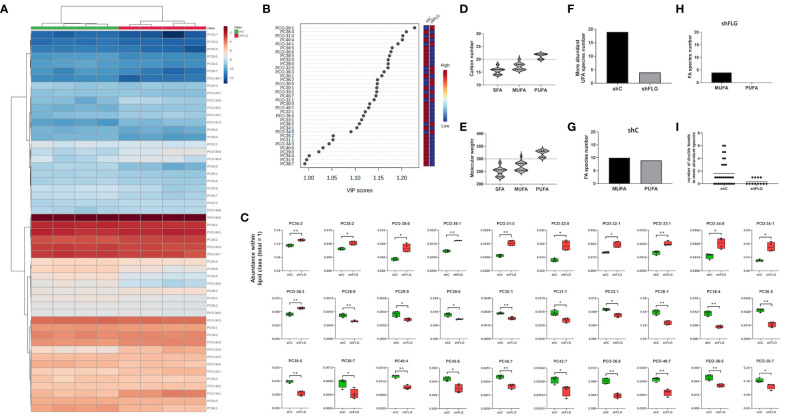
A filaggrin insufficiency background alters the landscape of the PLA2-digestible lipidome in keratinocytes. **(A–C)** Lipidomic analysis of PLA2-digestible lipid species in shC and shFLG keratinocytes; **(A)** heatmap of all detected lipid species; **(B)** lipid species most affected by filaggrin insufficiency accordingly to the PLS-DA analysis; the variance importance for the projection values (VIP) were used to sort lipids accordingly to their contribution to PLS-DA model; **(C)** boxplots showing lipid species significantly different in abundance; n = four biological replicates; unpaired t-test; FDR correction; **(D, E)** Fatty acid composition of differentially abundant phospholipids in keratinocytes by **(D)** chain length and **(E)** molecular weight of fatty acids; dotted line shows the size and mass benchmarks for optimal CD1a-mediated responses; **(F)** Number of the more abundant lipid species in keratinocytes; **(G, H)** UFAs represented in **(F)** found in either shC **(G)** or shFLG **(H)** keratinocytes by degree of unsaturation; **(I)** Number of double bonds in the more abundant UFA species in keratinocytes; PLA2, phospholipase A2; PC, diacylglycerophosphocholine; PCO, ether-linked glycerophosphocholine; PEO, ether-linked glycerophosphoethanoloamine; SFA, saturated fatty acid; UFA, unsaturated fatty acid; MUFA, monounsaturated fatty acid; PUFA, polyunsaturated fatty acid. *, p<0.05; **, p<0.01.

**Figure 7:**
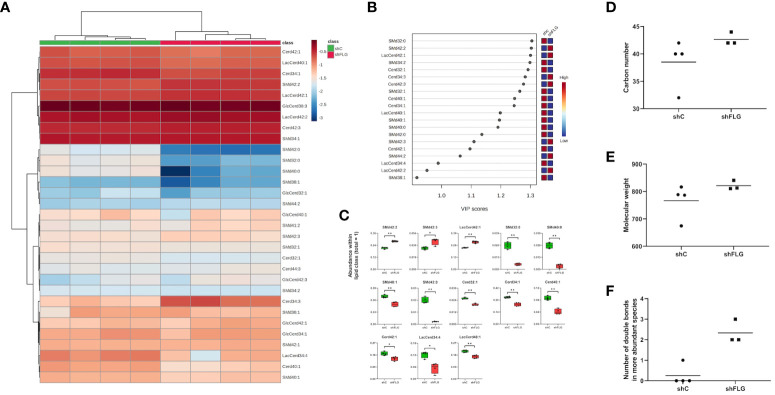
A filaggrin insufficiency background alters the landscape of non-PLA2-digestible lipidome in keratinocytes. **(A–C)** Lipidomic analysis of PLA2-nondigestible lipid species in keratinocytes; **(A)** heatmap of all detected lipid species; **(B)** lipid species most affected by filaggrin insufficiency accordingly to the PLS-DA analysis; the variance importance for the projection values (VIP) were used to sort lipids accordingly to their contribution to PLS-DA model; **(C)** boxplots showing lipid species significantly different in abundance; data for n=4 biological replicates, unpaired t-test, FDR correction; **(D–F)** Differentially abundant sphingomyelin species represented by combined sphingosine and fatty acid chain length; **(D)**, molecular weight **(E)** and a number of double bonds **(F)**; SMd, sphingomyelin; Cerd, ceramide; LacCerd, lactosylceramide; GlcCerd, glucosylceramide. *, p<0.05; **, p<0.01.

Finally, to understand the reasons behind the differential enrichment of permissive and non-permissive ligands in shFLG_sEV_ vs. shC_sEV_, we determine if the observed alterations reflected changes in the overall cellular lipid profile resulting from filaggrin insufficiency. Indeed, we observed remodeling of the PC composition in shFLG cells, with significantly lower content of very long-chain PUFAs (**Figures 6A–C**, **Supplementary Figure S7A**) in shFLG cells than in shC cells, corresponding to the alterations in sEV composition. Specifically, the content of the complex ether analogs of PCs, i.e., species containing long-chain polyunsaturated fatty acids (e.g., PCO40:7 and PCO36:6), was reduced in the shFLG cells in comparison to the shC cells, and shorter saturated or monounsaturated PCOs were dominant in filaggrin-insufficient cells (**Figure 6C**). Hence, similar to the sEVs, FAs identified as more abundant in the shC cells represented a much greater variety and closer match to the CD1a-response relevant carbon number and molecular weight benchmarks than those in shFLG cells, which was also true for fatty alcohols (FA-OHs) (**Figures 6D, E**). Interestingly, shC cells contained approximately four times more UFA species than shFLG keratinocytes (**Figure 6F**). Moreover, among the detected UFAs, a similar number of MUFA and PUFA species were detected in the shC cells, whereas in the shFLG cells, very few UFAs were MUFA species; no single PUFA was identified as being more abundant in these cells (**Figures 6G, H**). Finally, filaggrin-insufficient cells had a significantly reduced number of double bonds within the identified FAs and FA-OHs (**Figure 6I**). We also observed that filaggrin insufficiency significantly alters cellular content of several lipid species which are not PLA2 substates, namely ceramides (Cerds), lactosylceramides (LacCerds), and sphingomyelins (SMds) (**Figures 7A–C**, **Supplementary Figure S7B**). As for the differently abundant sphingomyelin species, we found that shFLG cells were enriched in sphingomyelins with longer chains and higher molecular mass compared to shC cells (**Figures 7D, E**). Moreover, all sphingomyelin species abundant in filaggrin-insufficient keratinocytes contained two or three double bonds within their chains, in contrast to the control cells, in which we found only one monosaturated sphingomyelin and all the remaining ones had completely saturated chains (**Figure 7F**). Finally, the two sphingomyelin species that contributed to the significant difference in the CD1a-dependent responses and were enriched in shFLG_sEV_, i.e., SMd 42:2 and 42:3, were also highly enriched in the filaggrin-knockdown cells.

### Dysregulated expression of enzymes involved in lipid metabolism on filaggrin-insufficiency background

**Figure 8:**
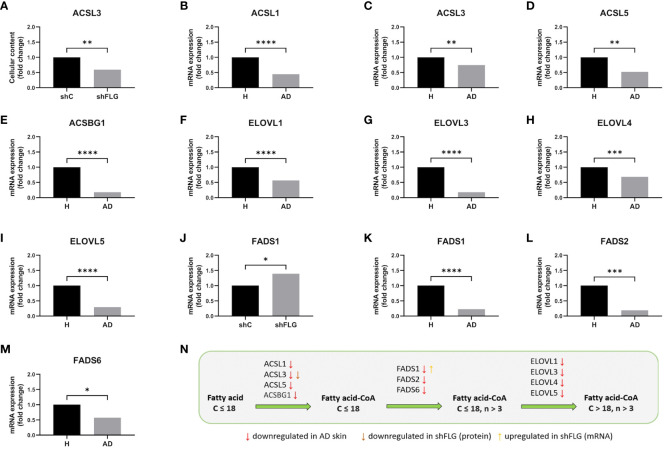
Filaggrin insufficiency in keratinocytes impacts enzymatic pathways of synthesis of lipids acting as substrates for generation of CD1a-dependent lipid neoantigens. **(A)** ACSL3 protein expression by cultured keratinocytes; n=4 biological replicates, unpaired t-test, Benjamini-Hochberg FDR; **(B-I)** Analysis of the data from Cole et al. (42) , showing the expression of **(B)** *ACSL1*; **(C)** *ACSL3*; **(D)** *ACSL5*; **(E)** *ACSBG*; **(F)** *ELOVL1*; **(G)** *ELOVL3*; **(H)** *ELOVL4*; **(I)** *ELOVL5* mRNA in AD skin; n=26 AD and n=10 healthy subjects; Benjamini-Hochberg FDR; **(J)** *FADS1* mRNA expression in cultured keratinocytes; n=3 biological replicates, t-test; **(K–M)** Analysis of the data from Cole et al. (42) , showing the expression of **(K)** *FADS1*; **(L)** *FADS2*; **(M)** *FADS6* mRNA in AD skin; n=26 AD and n=10 healthy subjects; all data are normalized to control (shC or H=1); **(N)** Summary of the changes in the lipid metabolic pathways identified in this study; simplified diagram; C, number of carbon atoms; n, number of double bonds; *, p<0.05; **, p<0.01; ***, p<0.001; ****, p<0.0001. *ACSL3*, long-chain-fatty-acid–CoA ligase 3; *ACSL1*, long-chain-fatty-acid–CoA ligase 1; *ACSL5*, long-chain-fatty-acid–CoA ligase 5; *ACSBG1*, long-chain-fatty-acid–CoA ligase *ACSBG1*; *ELOVL1*, *ELOVL* fatty acid elongase 1; *ELOVL3*, *ELOVL* fatty acid elongase 3; *ELOVL4*, *ELOVL* fatty acid elongase 4; *FADS1*, fatty acid desaturase 1; *FADS2*, fatty acid desaturase 2; *FADS6*, fatty acid desaturase 6.

Alteration in the skin lipid content and dysregulation of the lipid metabolic pathways was previously observed in the AD skin (42, 50, 51). Given the extent of the changes we detected, affecting multiple lipid classes both in the shFLG cells and their sEV compartments, we envisaged that the mechanism contributing to the phenotype would likely involve a pathway(s) with a major role in lipid metabolism and membrane formation. To this end, we identified that the long-chain-fatty-acid-CoA ligase 3 (ACSL3), implicated in free fatty acid conversion to activated acyl-CoA esters (52, 53), which is crucial in the membrane phospholipid synthesis process (54), is substantially downregulated in shFLG cells (**Figure 8A**). Interestingly, apart from ACSL3, we found additional isoforms of this enzyme to be downregulated in AD skin (at the mRNA level; **Figures 8B–E**). In contrast, the enzymes of the elongation of very long (ELOVL) fatty acid family, proposed to be involved in the process of fatty acid extension for CD1a ligands (48), were not differentially expressed in the *in vitro* models, whereas we observed downregulation of *ELOVL1, ELOVL3, ELOVL4*, and *ELOVL5* mRNA in AD skin (**Figures 8F–I**). In addition, we observed upregulation of the *FADS1* mRNA expression in the cells (**Figure 8J**), likely due to a compensatory nature in the 2D model, but downregulation of the *FADS1, FADS2*, and *FADS6* mRNA in the skin of patients with AD, suggesting more complex regulation of these enzymes during the stratification process (**Figures 8K–N**).

## Discussion

In this study, we present the first evidence that secretory vesicles may constitute an efficient source of lipid antigens for CD1-mediated presentation pathways and modulate T-cell responses. Specifically, we showed that sEVs are not immunologically inert in this system but may supply PLA2 substrates to modulate the activity of CD1a-specific T cells. We investigated this in the context of atopic dermatitis (AD) and the insufficient expression of the multifunctional epidermal barrier protein filaggrin. Loss-of-function mutations in *FLG* constitute the most prominent genetic predisposition factor for this disease (19), highlighting the role of filaggrin in supporting epidermal barrier function and controlling keratinocyte differentiation. Reduced filaggrin expression in the skin of AD patients and experimental models affects numerous processes that are hallmarks of effective epidermal differentiation and cornification (55), such as remodeling of the cytoskeleton (56), formation of tight junctions (57), lipid production (56), and changes in enzymatic activity (25, 58). *FLG* null mutations predispose individuals to microbial dysbiosis (59) and reduced ability to control skin infections, resulting in *S. aureus* superinfections (60) and a predisposition to eczema herpeticum (61). In this study, we used a knockdown model to mimic the downregulation of filaggrin expression dependent on the isolated inherited factor, which allowed us to dissect out the impact of AD inflammatory mediators and environmental factors that we are currently investigating in a separate study. The use of a stable knockdown line allowed us to overcome the low EV output from primary keratinocytes and the limited size of the AD skin samples. Our study, integrating the findings from 2D *in vitro* models with 3D organotypic cultures constructed of primary keratinocytes with the AD skin dataset, visualized the extent of changes resulting from filaggrin insufficiency and identified compartmental remodeling with relevance to immunological processes.

The initial results obtained in our study indicated that the addition of keratinocyte-derived sEVs did not affect peptide antigen processing or class I/II loading pathways, which could affect antigen presentation to T cells, regardless of the filaggrin status in the cells being a source of sEVs. The involvement of keratinocyte-derived sEVs in antigen-specific responses was previously studied by Kotzerke et al. in the context of peptide-specific responses to ovalbumin (OVA) in a murine model that failed to detect any apparent T-cell activation of OVA-specific T cells (11). However, the authors did not investigate filaggrin-insufficient mice or the perspective of lipid-specific responses; at the same time, significant differences in the CD1 system between the species (CD1a-c are absent in rodents) would hamper detection of any such responses, unless a humanized model was used. Recent work, which described *S. aureus* enterotoxin B sEV-mediated transfer from keratinocytes following superantigen exposure, described a potential for non-specific T-cell activation (62).

Here, we showed that sEVs contain CD1a lipid ligands with either activatory or inhibitory potential, judged with respect to IFNγ responses; most lipids supplied to T cells within sEV are permissive but weak ligands, corresponding to the autoantigen characteristics and demonstrating interindividual variability. We also found that some of the lipids may promote type 2 (pro-allergic) bias and stimulate IL-13 secretion from T cells. Interestingly, we found that filaggrin insufficiency reduced the capacity of sEVs to carry substrates suitable for the generation of type 1 response-inducing CD1a ligands. Given that sEV membranes contain a mixture of permissive and non-permissive lipids, a shift between type 1 and type 2 responses may reflect changes in the overall avidity during CD1a-mediated presentation to T cells. Specifically, it has been shown for both peptides and lipid presentation within the CD1d pathway that changes in ligand affinity (hence the overall interaction avidity) result in varied contact time between the cells and their activation level, leading to differential responses (63–66), i.e., the longer the time, the more type 1 bias. This “structure–activity relationship” has been proposed to result in a ligand-specific “cytokine fingerprint” (67, 68). Hence, the more biased towards type 2 responses observed in our study may result from the increased abundance of non-permissive ligands, disruption of the CD1a-TCR contact zone, and reduced T-cell interaction time leading to an altered T-cell activation profile.

In the context of atopic skin disease, we observed an extensive impact of filaggrin insufficiency on keratinocytes as a whole and their sEV compartment specifically. In addition, the loss of control of PLA2 activity (5) in the filaggrin insufficiency scenario may lead to even greater dominance of the higher-affinity inhibitory ligands released from sEVs to compound skin inflammation. Of note, although we did not observe any differential outcome in the class I/class II presentation pathways by simple addition of sEVs during antigen pulsation, it is still possible that sEVs from keratinocytes with insufficient filaggrin may exert additional relevant effects, e.g., through their altered propensity to interact with a recipient cell or undergo cellular uptake. This could be especially interesting in relation to cargo transfer to antigen-presenting cells, with a potential impact on the ability of sEVs to supply pathogen-associated molecular pattern compounds (PAMPs) or antigens for downstream activation of innate and adaptive immune responses, respectively.

Aberrant keratinocyte differentiation resulting from filaggrin insufficiency has previously been shown to contain a broad lipid dysregulation component *in vitro* (24) which correspond to the lipid abnormalities previously reported in AD skin *in vivo* (50, 51, 69). Here we determined that the altered sEV FA composition in our model of filaggrin-insufficient keratinocytes is a likely consequence of a reduction in the expression of enzymes in the long-chain fatty acyl-CoA ligase family (ACSLs), ELOVL elongases, and FAD desaturases. ACSLs are enzymes upstream of several critical cellular lipid metabolism pathways (53) that catalyze the process of fatty acid activation and the formation of fatty acyl-CoA esters, which regulate diverse cellular functions such as gene regulation, enzyme inhibition, modulation of ion channel function, and membrane fusion (52). ACSLs are implicated in membrane phospholipid biosynthesis; their involvement in the process of incorporation of MUFA and PUFA species into membrane phospholipids has been previously described for multiple ACSLs (54, 70, 71)*;* they also have a preference for polyunsaturated fatty acids (54, 70–72). An increase in saturated fatty acids and a decrease in polyunsaturated fatty acid content have been observed in rat hepatocytes in an ACSL3 knockdown model (70). As for allergic manifestations, methylation of the *ACSL3* 5’-CGI has been found to correlate with asthma status in children (73) and has been reported to increase in an allergen-induced airway hyperreactivity model in mice (74). Furthermore, methylation of *ACSL3* has also been identified as a signature predictive of clinical food allergy in children (75). Interestingly, this enzyme was also found in sEVs isolated from colostrum but not in mature breast milk (76). In our study, it was not detected in keratinocyte-derived sEVs, but could have resulted from the detection threshold.

It has been suggested that the elongation of very long (ELOVL) fatty acid enzymes, which control the length of very long fatty acids, may be involved in the generation of the long-chain sphingomyelins, such as 42:2. While there was no differential expression in our *in vitro* dataset, we and others have identified a decrease in *ELOVL* mRNA expression in AD skin (69). Upregulation of FADS1, which we believe may be a secondary compensatory mechanism (77), was the only additional finding relevant to this pathway in cultured keratinocytes. In contrast, mRNA for several FADS enzymes was downregulated in the AD skin but not in the organotypic model, which may suggest a more complex regulation where the inflammatory *milieu* may play an important role.

The downregulated expression of all these enzymes in the background of filaggrin insufficiency has important immunological consequences; we show that the lipid content in secreted sEVs is affected to the extent that it abolishes their capacity to provide substrates for the generation of the CD1a permissive self-antigens by PLA2, which provide homeostatic T-cell activation, contributing to tissue integrity. It has been previously determined that the optimal length of the lipid chain appropriate for accommodation within the CD1a groove is approximately 20 carbon atoms, and unsaturated lipids induce a superior response (45). Interestingly, when we compared responses obtained from the selected lipids found within the sEVs, it was not always the case, i.e., while we could see the highest level of responses to the polyunsaturated long C22:6 DHA, only some donors responded to this lipid; responses to C14:0 SFA were lower but more prevalent, while responses to Lyso-PC18:0 were less persistent over time.

While we did not find any changes in the sphingomyelin synthesis pathway, studies focusing on the loss of ACSL activity provide additional insight. Specifically, ACSL has been shown to regulate the composition of fatty acids and membrane lipids in lipid rafts (78) by the effect on ceramide expression, e.g., silencing of the enzyme results in the accumulation of ceramides and sphingomyelin analog in Drosophila (phosphoethanolamine ceramide; CerPE) (78, 79). Therefore, while the expression of the enzymes in the sphingolipid synthesis pathway may not be directly affected by filaggrin insufficiency, the increased supply of the substrates channelled into the ceramide/sphingolipid synthesis pathway is a very likely explanation for the accumulation of non-permissive sphingomyelins (80).

The skin is enriched in CD1a^+^ Langerhans cells, abundant in the epidermis (81, 82), CD1a is also inducibly expressed by dendritic cell populations deeper in the tissue (83, 84). Our findings are highly relevant to immunological events and tissue integrity (85), since the CD1a-restricted population has been shown to contain many autoreactive T cells capable of sensing barrier damage and promoting mechanisms engaged in tissue repair (46). CD1a-resticted responses also contribute to the control of pathogenic skin bacteria (6) and there seems to be an indication of their importance in the lungs and gut (86, 87), where CD1a-expressing cells can also be found (88–94). To this end, CD1a-restricted responses have been shown in a humanized model of *M. tuberculosis* infection (7) and in a range of *M. tuberculosis* lipopeptide (DMM) isomers (95). Our study determined that neoantigens derived from normal keratinocytes (filaggrin sufficient; replicated by shC_sEV_ in our study) are likely to be CD1a permissive ligands promoting autoreactive responses; their provision may support homeostasis at the skin barrier site or potentially even play an adjuvant-like role in antimicrobial immunity (96). In contrast, sEVs secreted in the filaggrin insufficiency background, containing altered lipid content, can inhibit type 1 T-cell responses and promote type 2 bias. Given the preference of the CD1a molecule to bind high-affinity inhibitory ligands (48), such as those contained within the sEVs produced by filaggrin-insufficient keratinocytes, their presence in the *milieu* would likely affect both low-level homeostatic and much more pronounced antimicrobial CD1a-mediated T-cell responses.

The data obtained by us with intact sEVs derived from keratinocytes do not provide enough readily available CD1a ligands, since sEVs do not drive marked T-cell reactivity in the absence of PLA2, thereby, reducing the risk of inflammation in the absence of an external threat. We believe that the mechanism we have identified does not seem to depend on the uptake/internalization of sEVs *per se*, given the results obtained with undigested sEVs. Specifically, in contrast to antigens within the intraluminal cargo, sEV internalization does not necessarily ensure the presentation of lipids enclosed within their membranes. This could be because sEVs, which are mainly phagocytosed (97), end up as phagolysosomes and therefore do not localize to the CD1a trafficking compartments. Alternatively, the lipids may remain sequestered within the membranes (either with the plasma membrane or EE membranes) after fusion and remain inaccessible to CD1a. It is important to note that allergens (5) and pathogens may constitute (59, 60, 98) a source of phospholipase A2 activity, either directly (99–101) or indirectly (6, 102, 103), and any additional impact of these external factors may also add another layer of complexity to this system. Simultaneously, normal keratinocyte-derived sEVs could potentially quench the toxic impact of PLA2 on cellular membranes, protecting the body from excess tissue damage during inflammation. sEVs can also shield commensal bacteria, which seem to be more susceptible to PLA2 than pathogenic strains (104). Since keratinocyte-derived sEVs transfer into the circulation and peripheral tissues, we hypothesized that the impact could potentially extend beyond the local tissue environment and promote chronic inflammation and Th2 bias underlying additional allergic manifestations in atopic patients. Given the evolutionary interspecies differences in communication in the skin tissue and possible systemic consequences would ideally be tested in a follow-up *in vivo* study, with the use of filaggrin-deficient animals generated on the CD1a humanized transgenic mice background. This would allow determination of the importance of the tissues beyond the skin, since a causative role of dysbiosis (105, 106), and chronic inflammation preceding the development or exacerbations in allergic asthma (107, 108), intestinal tissue damage (109, 110), and food allergy (111), affecting the development of tolerance to the encountered allergens (112) has been previously established; however, the precise role of the sEV-mediated transfer of lipid antigens would have to be experimentally confirmed.

In summary, we have shown that small secreted extracellular vesicles constitute a source of antigens for lipid presentation pathways and are actively involved in CD1a-mediated T-cell responses. We also established that cellular lipid metabolism, i.e., fatty acid elongation and desaturation pathways, determines the downstream outcome of these responses. In the case of AD, altered sEV lipidome can be traced back to aberrant keratinocyte differentiation, observed on filaggrin insufficiency background apparent both *in vitro* and in the skin of patients with AD. The resulting sEV lipid repertoire supports immune consequences, such as persistent allergic inflammation and dysbiosis in the AD skin.

## Data availability statement

The datasets presented in this study can be found in online repositories. The names of the repository/repositories and accession number(s) can be found below: GSE203409 (GEO) and PXD026859 (ProteomeXchange- PRIDE).

## Ethics statement

The studies involving humans were approved by Independent Bioethics Committee for Scientific Research at Medical University of Gdansk, ethical approval numbers: NKBBN/558/2017-2018 and 780 NKBBN/621-574/2020. The studies were conducted in accordance with the local legislation and institutional requirements. Buffy coats were obtained from blood donations from healthy donors the Regional Blood Centre in Gdansk. Written informed consent for participation was not required from the participants or the participants’ legal guardians/next of kin in accordance with the national legislation and institutional requirements.

## Author contributions

AK: Data curation, Formal Analysis, Investigation, Methodology, Validation, Visualization, Writing – original draft, Writing – review & editing. WH-B: Data curation, Formal Analysis, Investigation, Methodology, Validation, Writing – original draft, Writing – review & editing. JF: Formal Analysis, Investigation, Methodology, Validation, Visualization, Writing – review & editing. NK: Data curation, Investigation, Methodology, Writing – review & editing. LH: Data curation, Investigation, Methodology, Writing – review & editing, Formal Analysis. AB: Data curation, Formal Analysis, Investigation, Methodology, Validation, Writing – review & editing. RE: Methodology, Writing – review & editing, Investigation. AP: Methodology, Writing – review & editing, Investigation. ID: Methodology, Writing – review & editing, Investigation. SG: Formal Analysis, Methodology, Validation, Writing – review & editing. SB: Formal Analysis, Methodology, Validation, Writing – review & editing. GO: Conceptualization, Formal Analysis, Funding acquisition, Investigation, Methodology, Project administration, Supervision, Validation, Writing – review & editing. DG-O: Conceptualization, Data curation, Formal Analysis, Funding acquisition, Investigation, Methodology, Project administration, Resources, Supervision, Validation, Visualization, Writing – original draft, Writing – review & editing.
