# Supplementary material for: Keratinocyte-derived small extracellular vesicles supply antigens for CD1a-resticted T cells and promote their type 2 bias in the context of filaggrin insufficiency
Source: Front Immunol. 2024 Mar 22;15:1369238. doi: 10.3389/fimmu.2024.1369238 (PMC10995404; doi:10.3389/fimmu.2024.1369238)
Supplement: Supplementary file 1 [file DataSheet_1.pdf]

Supplementary figures

Figure S1.

siFLG organotypic skin model

Analysis type	Term	Fold enrichment	FDR
**Gene Ontology: Biological Process**	Antigen processing and presentation (GO:0019882)	2.16	$4.04*10^{-2}$
	Antigen processing and presentation of peptide antigen via MHC class I (GO:0002474)	2.81	$4.76*10^{-2}$
**Reactome Pathways**	Class I MHC mediated antigen processing & presentation (R-HSA-983169)	2.06	$2.75*10^{-3}$
	Antigen processing-Cross presentation (R-HSA-1236975)	3.24	$3.37*10^{-3}$

Fig. S1. Enrichment in the Gene Ontology and Reactome terms associated with antigen presentation identified within the siFLG organotypic skin model.

Proteome dataset analysed by Panther.

Figure S2.

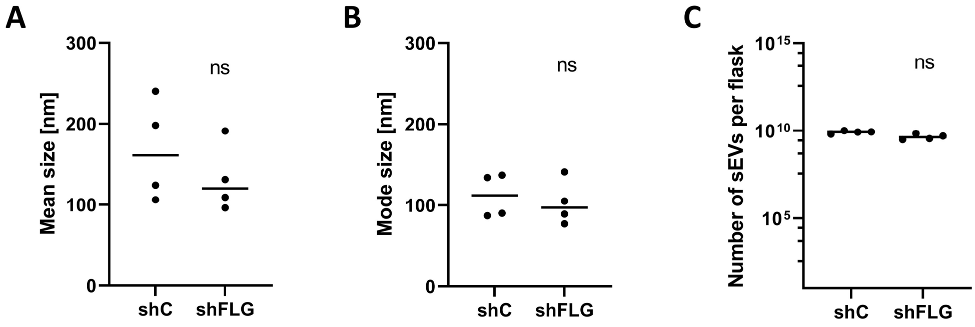

Fig. S2. Filaggrin insufficiency does not affect the size of sEVs or their production by keratinocytes.

(**A** and **B**) Size of keratinocyte-derived sEVs expressed as (A) mean and (B) mode measured by Nanoparticle Tracking Analysis (NTA); (**C**) Number of sEVs produced by keratinocytes per T75 cell culture flask measured by NTA. Combined data for n=4 biological replicates, paired t-test.

Figure S3.

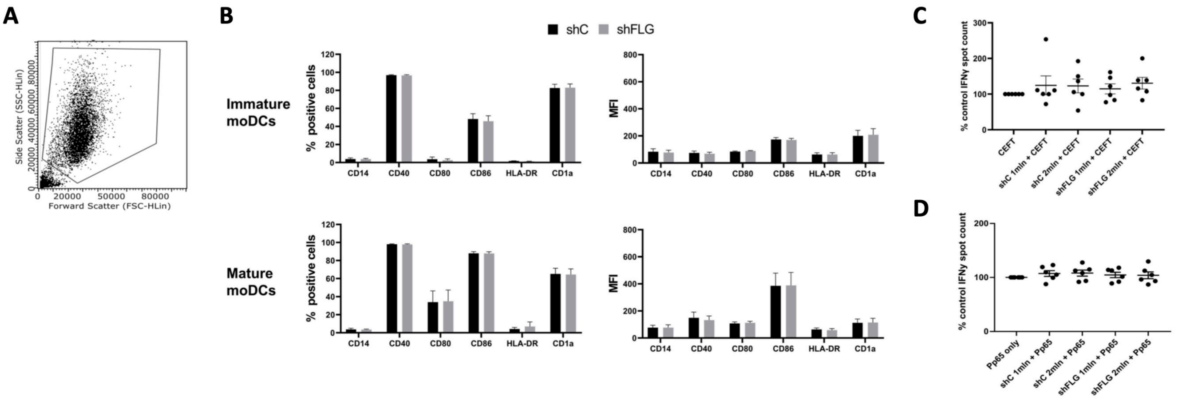

Fig. S3. sEVs secreted by keratinocytes do not impact T cell responses to peptide antigens.

(**A**) Flow cytometry gating strategy for MoDCs; (**B**) Expression of dendritic cell markers by immature and mature MoDCs treated with keratinocyte-derived sEVs at 10 µg/ml protein concentration (means +/- SEM are shown; combined data from n=5 donors; one-way ANOVA with Šídák's multiple comparisons test; (**C** to **D**) IFNγ production by T cells stimulated with immature moDCs pulsed with sEVs derived from 1 or 2 million keratinocytes and (C) CEFT peptides at 1 µg/ml per peptide or (D) 10 µg/ml of whole pp65 protein overnight measured by ELISpot assay (means +/- SEM are shown; data are normalized to control; data from n=6 donors; one-way ANOVA with Šídák's multiple comparisons test; moDC, monocyte-derived dendritic cell; MFI, mean fluorescence intensity.

Figure S4.

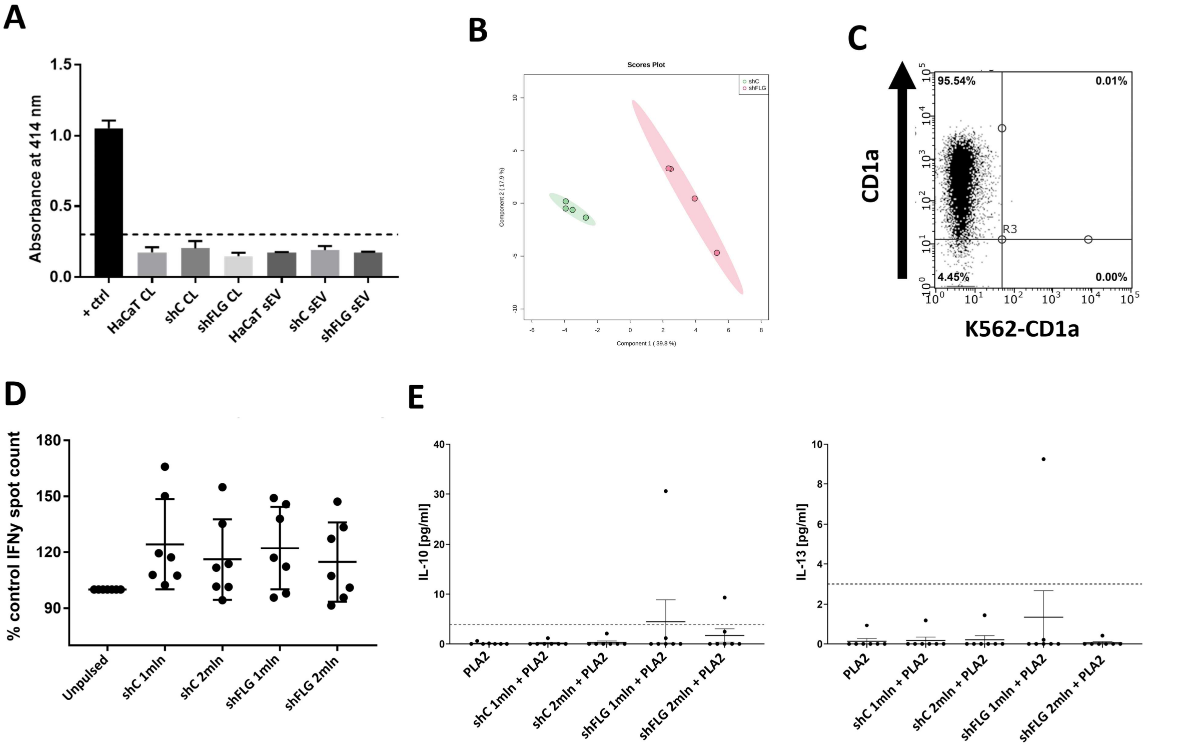

Fig. S4. Keratinocyte-derived sEVs are not a source of readily available CD1a antigens.

(**A**) cPLA2 activity measurement in keratinocytes and sEVs by a colorimetric assay (normalized to control; means from n=3 technical replicates; +/- SEM are shown; dashed line marks the limit of detection; (**B**) PLS-DA analysis of PLA2-digestible lipidome of sEVs; n=4 biological replicates; (**C**) CD1a expression by K562 cells CD1a transfectants; example flow cytometry data shown; (**D**) IFNγ responses from T cells stimulated with K562-CD1a cells pulsed with sEVs derived from 1 or 2 million keratinocytes overnight (means +/- SEM are shown; data are normalized to control=100%; n=7 donors; one-way ANOVA with Šídák's multiple comparisons test; (**E**) Production of IL-10 and IL-13 by T cells stimulated with K562-CD1a cells pulsed with sEVs from 1 or 2 million keratinocytes and 1 µg/ml PLA2 measured by ELISA; means +/- SEM are shown; n=7 donors; one-way ANOVA with Šídák's multiple comparisons test; dotted line marks the limit of detection; CL, cell lysate.

Figure S5.

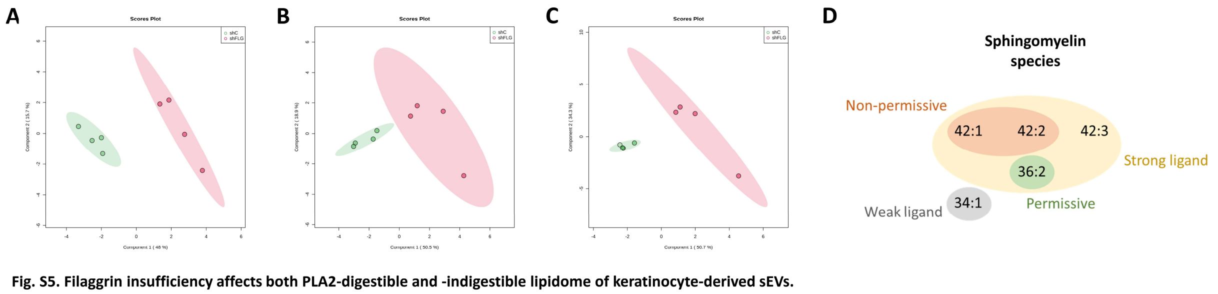

Fig. S5. Filaggrin insufficiency affects both PLA2-digestible and -indigestible lipidome of keratinocyte-derived sEVs.

(**A** and **C**) Score plot between PC1 and PC2 obtained from PLS-DA analysis of (A) glycerophosphocholine-related products of sEV digestion with 1 µg/ml PLA2 for 1h, (B) PLA2-non-digestible lipids in sEVs and (C) PLA2-non-digestible lipids in sEVs digested with 1 µg/ml PLA2 for 1h. (**D**) Characteristics of sphingomyelin species identified as potential CD1a antigens by Cotton *et al.*[38].

Figure S6.

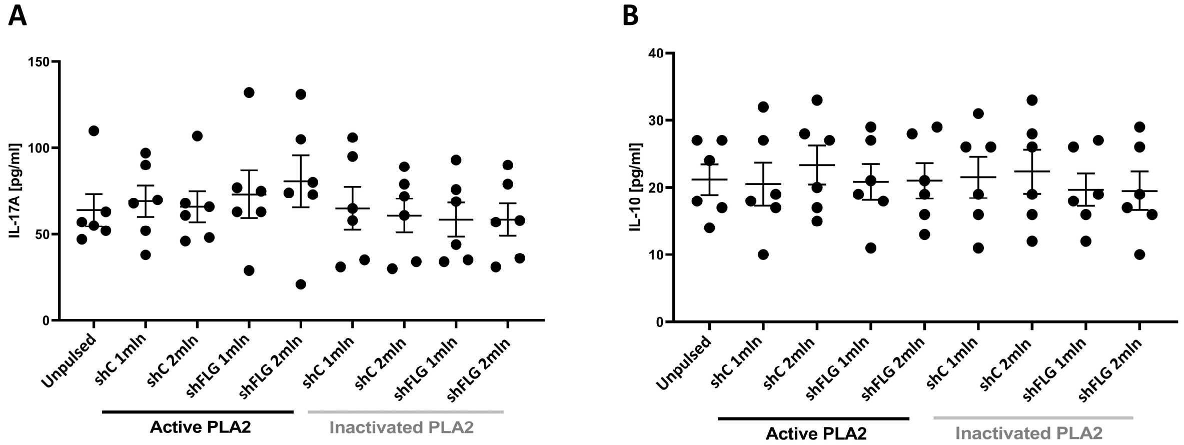

Fig. S6. PLA2-digested sEVs do not alter IL-10 or IL-17A production by T cells regardless of the keratinocyte filaggrin status.

(**A** and **B**) Production of (A) IL-17A and (B) IL-10 by T cells stimulated with K562-CD1a cells pulsed overnight with sEVs from 1 or 2 million keratinocytes digested with 1 μg/ml PLA2 for 1h (means +/- SEM shown, n=6 donors, one-way ANOVA with Šídák's multiple comparisons test).

Figure S7.

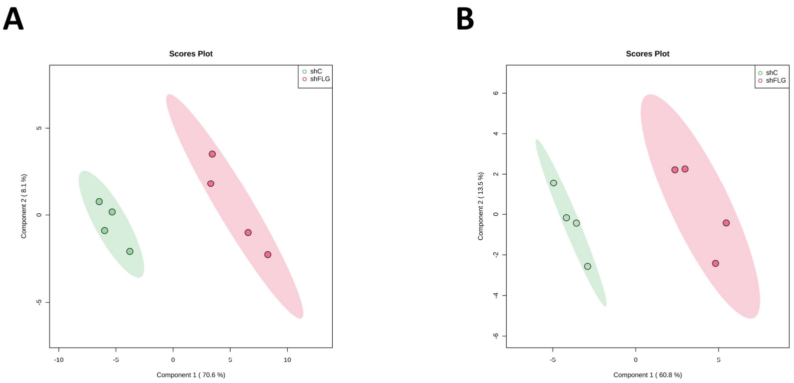

Fig. S7. Keratinocyte lipid composition is impacted by filaggrin insufficiency.

(**A** and **B**) Score plot between PC1 and PC2 obtained from PLS-DA analysis of keratinocyte lipidome; (A) PLA2-digestible and (B) -non-digestible lipid species.
